# Undesired Small RNAs Originate from an Artificial microRNA Precursor in Transgenic Petunia (*Petunia hybrida*)

**DOI:** 10.1371/journal.pone.0098783

**Published:** 2014-06-04

**Authors:** Yulong Guo, Yao Han, Jing Ma, Huiping Wang, Xianchun Sang, Mingyang Li

**Affiliations:** 1 Chongqing Engineering Research Center for Floriculture, Key Laboratory of Horticulture Science for Southern Mountainous Regions, Ministry of Education, College of Horticulture and Landscape Architecture, Southwest University, Chongqing, China; 2 College of Agronomy and Biotechnology, Southwest University, Chongqing, China; CNRS UMR7622 & University Paris 6 Pierre-et-Marie-Curie, France

## Abstract

Although artificial microRNA (amiRNA) technology has been used frequently in gene silencing in plants, little research has been devoted to investigating the accuracy of amiRNA precursor processing. In this work, amiRNAchs1 (amiRchs1), based on the *Arabidopsis* miR319a precursor, was expressed in order to suppress the expression of *CHS* genes in petunia. The transgenic plants showed the *CHS* gene-silencing phenotype. A modified 5′ RACE technique was used to map small-RNA-directed cleavage sites and to detect processing intermediates of the amiRchs1 precursor. The results showed that the target *CHS* mRNAs were cut at the expected sites and that the amiRchs1 precursor was processed from loop to base. The accumulation of small RNAs in amiRchs1 transgenic petunia petals was analyzed using the deep-sequencing technique. The results showed that, alongside the accumulation of the desired artificial microRNAs, additional small RNAs that originated from other regions of the amiRNA precursor were also accumulated at high frequency. Some of these had previously been found to be accumulated at low frequency in the products of ath-miR319a precursor processing and some of them were accompanied by 3′-tailing variant. Potential targets of the undesired small RNAs were discovered in petunia and other Solanaceae plants. The findings draw attention to the potential occurrence of undesired target silencing induced by such additional small RNAs when amiRNA technology is used. No appreciable production of secondary small RNAs occurred, despite the fact that amiRchs1 was designed to have perfect complementarity to its *CHS-J* target. This confirmed that perfect pairing between an amiRNA and its targets is not the trigger for secondary small RNA production. In conjunction with the observation that amiRNAs with perfect complementarity to their target genes show high efficiency and specificity in gene silencing, this finding has an important bearing on future applications of amiRNAs in gene silencing in plants.

## Introduction

MicroRNAs (miRNAs) are a class of small RNAs of around 21 nt (nucleotides) in length, which are generated from imperfect fold-back regions of long endogenous primary transcripts (pri-miRNAs). In plants, miRNAs repress gene expression at the transcriptional, post-transcriptional and translational levels [1]. They play pivotal roles in plant development [2], [3], and are also involved in a range of other biological functions including hormonal regulation [4], [5], nutrient homeostasis [6], [7] and responses to various biotic and abiotic stresses [8]–[10].

Most plant miRNAs are transcribed by RNA polymerase II [11]. After transcription, the newly formed pri-miRNA transcripts must be capped and polyadenylated to promote stabilization, and sometimes they must also be spliced to promote the formation of stem-loop structural features [12]. The pri-miRNAs are then processed by DICER-LIKE (DCL) RNAaseIII endonucleases into short miRNA/miRNA^*^ duplexes with 2nt 3′-overhangs. In *Arabidopsis*, pri-miRNA processing is mainly orchestrated by DCL1, with the assistance of the dsRNA-binding protein, HYPONASTIC LEAVES1 (HYL1), and the C2H2-zinc finger protein, SERRATE, to improve the efficiency and precision of cleavage. DCL1 and HYL1, together with other associated factors, are co-localized in sub-nuclear regions termed Dicing-bodies (D-bodies), where miRNAs are processed [13], [14]. The biogenesis of most animal and plant miRNAs usually begins with a cut at the base of their stem-loop structures, thereby yielding precursors (pre-miRNAs), which are then further cut to produce 21–22 nt miRNA duplexes [15]. RNA secondary structure affects both the accuracy and the productivity of plant miRNA processing. For the processing of *Arabidopsis* pri-miR172a, the 14- to 15-base-pair stem region below the miRNA/miRNA^*^ duplex is essential, although small unpaired bulges that do not damage its linear structure are tolerated; and a loop is required, although mutations in the terminal loop are mostly neutral [16], [17]. The structural features of most conserved plant pri-miRNAs are similar to those of pri-miR172a and thus it is assumed that they have a similar processing mechanism [16], [18]. However, for the ‘long fold-back’ pri-miRNAs, pri-miR159 and pri-miR319, the processing mechanism is different from that undergone by animal miRNAs and most plant conserved miRNAs; it begins with a cleavage next to the terminal loop, and then DCL1 cuts three more times at 20– 22 nt intervals until the miRNA/miRNA^*^ duplex is released [19]. In contrast to the processing of pri-miR172a, the precursor sequences below the miRNA/miRNA^*^ duplex are dispensable for pri-miR319 processing, but the conserved upper stem is critical [19].

 Following their release from D-bodies, the miRNA/miRNA^*^ duplexes are stabilized by the addition of a methyl group at their 3′-end, catalyzed by the methyltransferase protein, HUA ENHANCER1 [20]. Subsequently, the miRNA strands are loaded onto ARGONAUTE-containing, RNA-induced silencing complexes (RISCs), and the miRNA^*^ strands are generally degraded. Plant miRNA-loaded RISCs recognize their target genes by highly complementary pairing between the miRNA and its target mRNA [1]; thus, a family of plant miRNAs can only repress the expression of a few target genes, usually duplicated genes. After a plant RISC has recognized its target mRNA, cleavage of the target at the central region of the predicted hybrid can usually be observed. Both the mechanism of action of cleavage and the sites at which it can occur have been validated in a range of studies using technologies such as 5′ RNA ligase-mediated rapid amplification of 5′ cDNA ends (5′ RLM-RACE) [21] and degradome sequencing [22].

In addition to resulting in mRNA degradation, the miRNA-mediated cleavage of target mRNAs can in some cases trigger the biogenesis of phased, secondary small interfering RNAs (phasiRNAs) [23], which can in turn silence additional genes, leading to a cascade of gene silencing.

A few classes of plant miRNA precursor have been successfully engineered to silence genes of interest, by replacing natural miRNAs with specifically modified or “designed” miRNA molecules, termed artificial miRNAs (amiRNAs) [24]–[28]. AmiRNAs engineered by modifications of *Arabidopsis MIR319a* precursor, the precursor backbone most commonly used, have been successfully used to induce gene silencing in *Arabidopsis* [29], tobacco [25], eggplant [30], soybean [31], *Medicago* [32], [33], and *Physcomitrella patens* [34]. Recently, this technology has also been used to silence genes in the vegetative cells of pollen in *Petunia inflata* [35]. When amiRNA gene silencing technology is used in plants, it is usually assumed that the amiRNA precursor is specifically processed to produce a single mature amiRNA [24]. Data relating to the processing of amiRNA precursors are scant, however, so the accuracy of amiRNA precursor processing requires further investigation.

In the study reported here, deep sequencing and modified 5′ RLM-RACE technology have been used to investigate the accuracy of amiRNA precursor processing in petunia. It is demonstrated that, in addition to the production of amiRNAs possessing the intended sequences, the processing of amiRNA precursors can lead to the abundant accumulation of additional small RNAs that may have potentially detrimental effects on unintended gene targets.

## Materials and Methods

### Plant materials

Inbred V26 *Petunia hybrid* was used as the recipient of amiRchs1 transgene construct. Inbred V26 was a generous gift from Prof. Manzhu Bao (Huazhong Agricultural University, China). Transgenic and wild type plants were grown side by side under a 16/8-h photoperiod in a greenhouse equipped with high-pressure sodium lights, at an intensity of 100–200 µmol·m^−2^·s^−1^.

### Production of transgenic plants expressing the CHS-amiRNA construct

At the start of this work, when it was decided to use amiRNA to suppress the expression of petunia genes, petunia had not been included in Web MicroRNA Designer (WMD) [24]. Thus, artificial microRNAchs (amiRchs) molecules were designed following the rules for amiRNA design introduced by Schwab et al. [24]: i.e. 21 nt long; a uridine at position 1 and an adenine at position 10; positions 2 and 12 to have no mismatch to the target; and the amiRNA to display 5′ instability relative to its corresponding miRNA^*^. The amiRNA candidate sequences designed in this way were then submitted to the RNAcofold WebServer (http://rna.tbi.univie.ac.at/cgi-bin/RNAcofold.cgi), in order to predict the free energy of formation of the amiRNA/target hybrid. One of the candidate amiRNA sequences, amiRchs1, was then selected for the production of the amiRNA silencing construct used for the investigation.

The amiRchs1 precursor was synthesized as described by Schwab et al. [24]. Routine molecular cloning procedures were used for plasmid construction, and the primers used are listed in Table 1. The *Arabidopsis* miR319a precursor (pRS300, a present from Prof. Detlef Weigel, Max Planck Institute for Developmental Biology, Tübingen, Germany) was used as the backbone for amiRchs1 construction and expression. An overlap PCR method was used to substitute the designed amiRchs1 sequence for the natural miRNA sequence and, at the same time, to modify the miRNA^*^ region. The PCR procedure was carried out using a Mastercycler 5331 (Eppendorf, Hamburg, Germany), using pRS300 plasmid DNA as the template, together with the oligonucleotide sequences I, II, III, IV and the general primers A and B. The PCR products were then cloned into pMD19-T (Takara, Dalian, China) and the resulting clones were sequenced, using T3 primer, in order to select clones that were free of errors introduced by PCR.

**Table 1:** PCR primers used in this study.

Name	Sequence(5′→3′)	Comments
amiRchs1-I	gatgttggtacatcatgagtcgctctctcttttgtattcc	amiRchs1 precursor synthesis
amiRchs1-II	gagcgactcatgatgtaccaacatcaaagagaatcaatga	amiRchs1 precursor synthesis
amiRchs1-III	gagcaactcatgatgaaccaacttcacaggtcgtgatatg	amiRchs1 precursor synthesis
amiRchs1-IV	gaagttggttcatcatgagttgctctacatatatattcct	amiRchs1 precursor synthesis
Primer A	ctgcaaggcgattaagttgggtaac	General primer for amiRNA precursor synthesis
Primer B	gcggataacaatttcacacaggaaacag	General primer for amiRNA precursor synthesis
qUBQ-F	tggaggatggaaggactttgg	qRT-PCR
qUBQ-R	caggacgacaacaagcaacag	qRT-PCR
qCHSA-F	ggcgcgatcattataggttc	qRT-PCR
qCHSA-R	tttgagatcagcccaggaac	qRT-PCR
qCHSJ-F	aaagtttagtggaggcattcc	qRT-PCR
qCHSJ-R	tccatactcactcaagacatg	qRT-PCR
CHSA5R-1	gtagttcctaaaccttctttggctgag	5′ RACE (round 1) to map cleavage sites
CHSA5R-2	tgagcaatccagaatagagagttccaa	5′ RACE (round 2) to map cleavage sites
CHSJ5R-1	agagacactatggagcacaacagtt	5′ RACE (round 1) to map cleavage sites
CHSJ5R-2	tagagttccagtcagaaatgcccaat	5′ RACE (round 2) to map cleavage sites
RACE5-1	cgactggagcacgaggacactga	General GeneRacer 5′ Primer (round 1)
RACE5-2	ggacactgacatggactgaaggagta	General GeneRacer 5′ Nested Primer (round 2)
amiRchs5R-1	tgagcgaaaccctataagaaccctaa	5′ RACE (round1) to map processing intermediates
amiRchs5R-2	acgaaggcagcatatatgtcacttag	5′ RACE (round2) to map processing intermediates

The amiRchs1 precursor fragment was released by the use of *Pst*I and *Bam*HI and then cloned into an intermediate vector under the control of 2×35S promoters and a 35S poly-A sequence, which were amplified from vector pSAT3 [36]. The expression box containing the amiRchs1 precursor sequence was then released using *Sal*1 and *Bam*H1 and inserted into the multiple cloning site (MCS) of pCAMBIA2301 to produce the pCMF-amiRchs1 vector. The resultant vector was then introduced into *Agrobacterium tumefaciens* strain GV3101 by electroporation.

### Plant genetic transformation

Plant genetic transformation and the regeneration of transformants were performed as described by Jorgensen et al. [37]. Briefly, V26 leaf-discs were immersed in *Agrobacterium* suspension for about 5 min, blotted dry, plated on co-cultivation medium, and then incubated in darkness at 22°C for 48 h. The explants were then transferred to selection medium. Four weeks later, callus islets were excised from the mother leaf-segments, and sub-cultured separately. When adventitious shoots appeared, the shoots regenerated from each callus islet were considered to constitute an independent transformation line. The shoots were excised and sub-cultured on rooting medium. All media used were as reported by Jorgensen et al. [37].

### Total RNA extraction and real-time RT-PCR analysis

**Figure 1:**
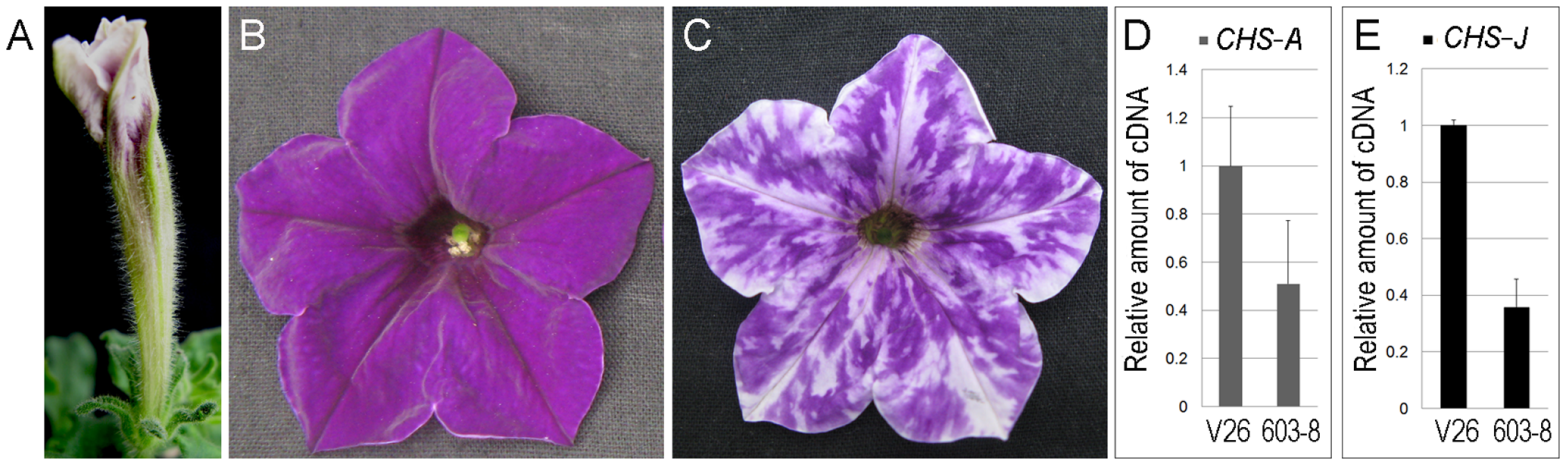
Phenotype of amiRchs1 transgenic flowers. (A) Opening flower bud of amiRchs1 transgenic plants (total RNA was extracted at this stage). (B) V26 (wild-type) flower. (C) amiRchs1 transgenic flower. (D–E) qRT-PCR detection of mRNA levels of the *CHS-A* (D) and *CHS-J* (E) gene in V26 and amiRchs1 transgenic petals. Data were normalized against petunia ubiquitin gene and were means of three biological pools (each with three technical replicates); the error bars indicate SD.

Total RNA was extracted from the petals of opening flower buds of V26 and transgenic plants (Figure 1A). Tissues were frozen with liquid nitrogen and extracted with TRIzol Reagent (Invitrogen, Carlsbad, CA, USA), used according to the manufacturer's directions. The concentration and quality of total RNA were analyzed using a NanoDrop 2000 spectrophotometer (Thermo Scientific, Waltham, MA, USA) and by gel electrophoresis. Total RNA was treated with RNase-free DNase I (Roche, Penzberg, Germany) and then reverse-transcribed to cDNA using PrimeScipt RT Master Mix (Takara). Specific primers (qCHSA-F and qCHSA-R, qCHSJ-F and qCHSJ-R, Table 1) for the amplification of *CHS-A* or *CHS-J* were synthesized as reported by Koseki et al [38]. Primers for the amplification of *UBQ* (qUBQ-F and qUBQ-R, Table 1) were synthesized according to Mallona et al. [39]. Each qRT- PCR reaction was performed in a 10 µL volume containing 0.5 µL of cDNA, 0.5 µL of each primer (10 µmol/L) and 5 µL of 2×SsoFast EvaGreen Supermix (Bio-Rad, Hercules, CA, USA), and was carried out on a CFX96 Real-time PCR Detection System (Bio-Rad) under the following conditions: 95°C for 30 s, followed by 39 cycles (each of 95°C for 5 s, then 60°C for 5 s), followed by melt curve analysis. The data were normalized using ubiquitin (*UBQ*) as endogenous control and analyzed to calculate relative expression values (ΔΔ*Ct* mode) as described by Schmittgenl and Livak [40]. Three technical and three biological replicates were performed for each sample and the standard deviation was calculated.

### Cleavage site mapping

To map the cleavage sites of the *CHS-A* and *CHS-J* target mRNAs, a modified procedure for 5′ RLM-RACE was carried out using the GeneRacer kit (Invitrogen), as described previously [21]. Five micrograms of total RNA from wild-type and amiRchs1 transgenic petals, respectively (without any prior treatment with calf intestine alkaline phosphatase and tobacco acid pyrophosphatase), were ligated to the adapter for 5′ RLM-RACE. Amplification was carried out as described in the manufacturer's instruction manual. The initial PCR (round 1) was performed using the 5′ RACE outer primer (RACE5-1, Table 1) from the manufacturer and a gene-specific outer primer (CHSA5R-1 or CHSJ5R-1, Table 1). Nested PCR (round 2) was performed using 1/50 of the initial PCR products as the template, together with GeneRacer 5′ Nested Primer (RACE5-2, Table 1) and a gene-specific primer (CHSA5R-2 or CHSJ5R-2, Table 1). The PCR products were cleaned using an AxyPrep PCR Clean-up kit (Axygen, Union City, CA, USA) and cloned using a pMD19-T cloning kit (Takara). DNA sequencing was undertaken by BGI (Genomics Institute of Science and Technology Co., Ltd, Shenzhen, China).

To detect the processing intermediates of the amiRchs1 precursor, the same procedure was used as described above for the mapping of the cleavage sites of the *CHS* target mRNAs. The gene-specific PCR primers were amiRchs5R-1 and amiRchs5R-2 (Table 1). For the detection of PCR-amplified fragments by gel electrophoresis, the PCR products were resolved on 10% (w/v) polyacrylamide gels and detected by silver staining.

### Deep-sequence analysis of small RNAs

One microgram of high-quality total RNA from petals of the transgenic line 603-8 was ligated to 5′- and 3′-adaptors, reverse transcribed, and then amplified by PCR (12 cycles), using a Truseq Small RNA sample preparation kit (Illumina, Santiago, CA, USA) according to the manufacturer's protocol. The library of small RNAs was purified by electrophoresis on a 6% Novex TBE PAGE gel (Invitrogen). Following quantification using TBS380 (Turner BioSystems, Sunnyvale, CA, USA), the nucleotide sequence of the amplified cDNA was analyzed using Illumina Hiseq 2000 (2×100 bp read length). The nucleotide sequence data have been deposited in the NCBI Sequence Read Archive under the accession number SRP036869. Only left side reads were used for analysis in this work.

The raw reads were trimmed by removing low-quality reads (Q value<20), adapter sequences, reads with ambiguous bases ‘N’, and fragments of less than 18 nt in length, using a Fastx-Toolkit (http://hannonlab.cshl.edu/fastx_toolkit/). The filtered small RNAs of 18–32 nt in length were then mapped onto the nucleotide sequences of the amiRchs1 precursor (allowing only perfect matches), and onto the *CHS-A* (GenBanK database accession X14591) and *CHS-J* (X14597) gene regions, respectively (allowing 4 mismatches).

Two libraries of Illumina SBS sequencing data [41] for small RNAs from i) the petals of line V26 (GSM433598) and from ii) the petals of transgenic V26 constitutively expressing the *CHS-A* coding sequence and displaying white (silenced) flowers (GSM346607) were downloaded from the Gene Expression Omnibus (GEO) database. They were analyzed in parallel. When necessary, small RNA data [42] obtained from the 454 Sequencing (http://www.petunia_smrna.leeds.ac.uk/) of petunia flower buds were searched.

### Small RNA target prediction

TargetSearch integrated into WMD3 Web Server (http://wmd3.weigelworld.org/) was used to search for potential targets of the undesired small RNAs identified in this study. TargetSearch is based on GenomeMapper (http://www.1001genomes.org), a sequence alignment tool. The search was carried out using default parameters (i.e., Mismatches: 5, Apply microRNA filter: yes, Perfect-match-dG cutoff: 70%, Hybridization temperature: 23°C, Folding program: RNAcofold, Direction: reverse, Allow gaps: no). Small RNAs of more than 19 nt in length were used for the search. In searching for targets in the *Petunia* genus, whole genomes were chosen, which included EST or transcript releases of *P. hybrida*, *P. axillaris* and *P. inflata* (*P. axillaris* and *P. inflata* are putative parents of *P. hybrida*). In searching for targets in tobacco, tomato and potato, EST releases of *Nicotiana tabacum* EST NtGI-7.0, *Solanum lycopersicum* EST LGI-13.0 and *Solanum tuberosum* EST StGI-13.0 were chosen, respectively.

## Results

### *CHS* gene silencing by an amiRNA in *Petunia hybrida*

**Figure 2:**
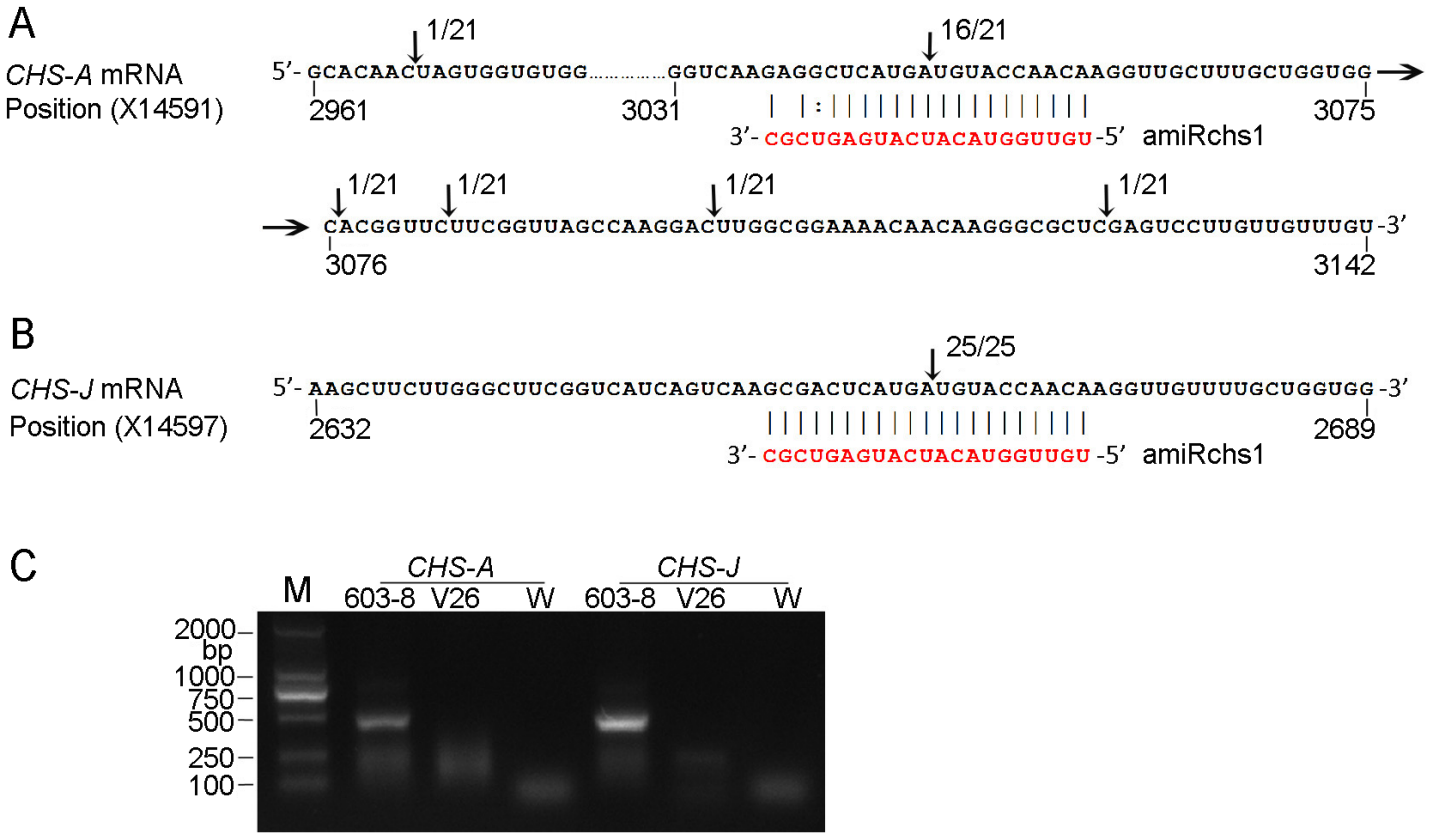
Mapping of target cleavage products by 5′ RLM-RACE. The cleavage sites and the number of sequenced clones corresponding to each site are indicated by arrows. For most of the sequenced clones, the 5′ end was at the expected position, opposite to nucleotides 10–11 of amiRchs1. (A) Cleavage of *CHS-A* mRNA. (B) Cleavage of *CHS-J* mRNA. (C) Agarose gel showing products after 5′ RLM-RACE PCR amplification. M, Marker; W, water.

**Figure 3:**
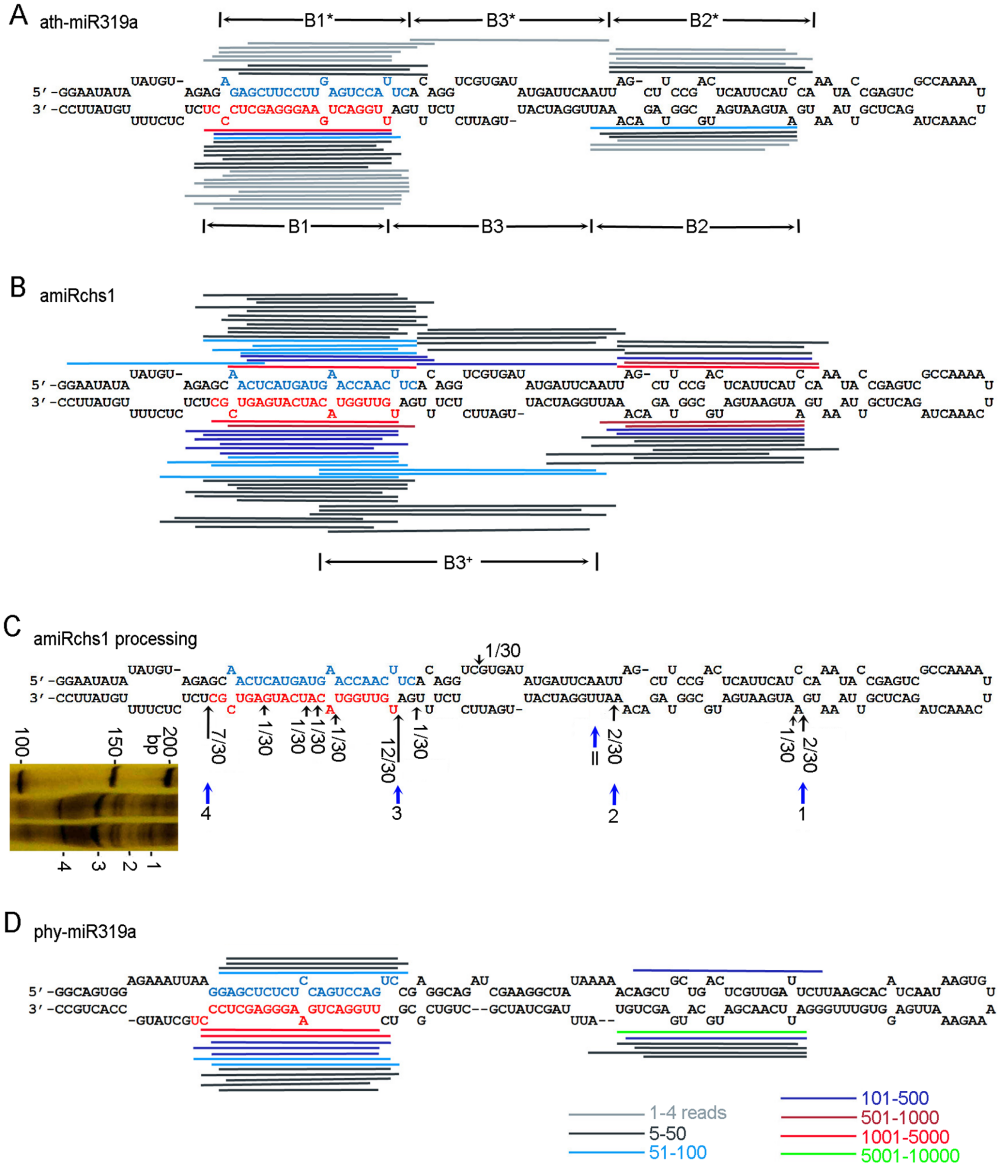
Processing of amiRchs1 and miR319 precursors. (A) Small RNA sequences from the miRBase database (v20 http://miRbase.org/index.shtml) were incorporated into the predicted stem-loop structure of the *Arabidopsis* miR319a precursor. Small RNAs cloned fewer than 5 times are also indicated. (B) Small RNA sequences from amiRchs1 transgenic petals incorporated into the scheme for the amiRchs1 precursor. Only small RNAs cloned more than 5 times are indicated. (C) Processing intermediates detected using 5′ RLM-RACE PCR amplification. The positions of cleavage sites, as revealed by 5′ RACE, and the number of sequenced clones corresponding to each site, are indicated by black arrows. The four sites marking the origins of B1 and B2 small RNAs, and corresponding to the four marked fragments in the polyacrylamide gel, are indicated by blue arrows (Sites 1, 2, 3 and 4). Site II corresponds to the cleavage site that marks the origin of the most frequent B1 small RNAs, but the processing intermediates had not been sampled by random sequencing of RACE PCR products. Left inset: Polyacrylamide gel showing fragments after 5′ RACE. (D) Small RNA sequences from amiRchs1 transgenic petals incorporated into the scheme for the petunia miR319a precursor. Only small RNAs cloned more than 5 times are indicated. B1, B1^*^, B2, and B2^*^ correspond to the regions producing the sequences previously designated as miR319a.1, miR319a.1^*^, miR319a.2 and miR319a.2^*^ [48], [49], respectively. B3 corresponds to the region between B1 and B2, B3^*^ corresponds to the region between B1^*^ to B2^*^, and B3^+^ corresponds to a region longer than B3, in which the 3′ ends of B3 sequences stretched into the middle of B1.

In order to suppress the expression of genes in petunia using an amiRNA, the amiRchs1 molecule was designed and its precursor was synthesized as described by Schwab et al. [24]. The engineered amiRchs1 was found to pair with *CHS-A* (X14591) from nt3037 to nt3057, with a mismatch and a G-U wobble at the 3′ end of amiRchs1, and a ΔG for heterodimer binding of −31.38 kcal/mol (Figure 2A). It paired perfectly with *CHS-J* (X14597) from nt2661 to nt2681, and the ΔG for heterodimer binding was −37.02 kcal/mol (Figure 2B). The secondary structure of the amiRchs1 precursor predicted by Mfold [43] was very similar to that found for the *Arabidopsis* miR319a precursor (Figure 3A and B). A difference between them was that an extra-base pair occurred below the miRNA/miRNA^*^ duplex in the amiRchs1 precursor (Figure 3A and B). This extra-base pair was introduced by replacing a 20 bp sequence in *MIR319a* with a 21bp designed sequence when amiRchs1 was engineered according to the procedure of Schwab et al [24], [44].

The amiRchs1 construct was introduced into V26 (Figure 1B and C) and two other genotypes (Figure S1). Transgenic lines showing altered flower color were produced in all three cases. Amongst the ten transgenic lines regenerated from V26, six lines produced flowers with conspicuous color alterations. Their petals contained randomly located white or pale sectors, or were nearly white. Because these transgenic plants displaying phenotypic changes were self-sterile, they were pollinated with V26 pollen. A line (603–8) that produced offspring in a ratio of approximately one wild-type plant to one mutant plant was used in this study (Figure 1C).

Two CHS genes (*CHS-A* and *CHS-J*) are active in petunia floral tissues, and the level of *CHS-A* gene expression is higher than that of *CHS-J* [45], [46]. The mRNA levels for both genes were analyzed in the petals of opening flower buds. For both *CHS-A* and *CHS-J*, mRNA accumulation was clearly reduced in the amiRchs1 transgenic line (Figure 1D and E), confirming the occurrence of *CHS-A* and *CHS-J* RNA degradation.

### AmiRchs1-directed cleavage of *CHS-A* and *CHS-J* mRNAs

Using *CHS-A* or *CHS-J* gene-specific primers respectively, 5′ RLM-RACE produced only one clear band (Figure 2C). In the case of *CHS-A*, sequencing results showed that of 23 clones, 21 mapped onto *CHS-A* (Figure 2A), and the other two were non-specific amplification products. Moreover, most of the *CHS-A* transcript cleavages occurred between the sites complementary to the 10th and 11th positions of the engineered (also identified by deep sequencing, see below) amiRchs1, although one cleavage occurred upstream of the most frequent cleavage site, and four occurred downstream; these may have represented aberrant mRNAs. In the case of *CHS-J*, of the 29 clones sequenced, 25 mapped to *CHS-J*. Cleavage occurred only between the sites complementary to the 10th and 11th positions of amiRchs1 (Figure 2B). These results indicated that the artificially synthesized amiRchs1 were effective in guiding the RISCs to their targets and that they resulted in cleavage at the predicted sites.

### Detecting processing intermediates of the amiRchs1 precursor by RACE

RACE PCR to detect processing intermediates of the amiRchs1 precursor generated more than four products, of different sizes (Figure 3C). Two of these products (bands 3 and 4) were clearly accumulated to a higher level than the others. Sequencing of the RACE products revealed that they corresponded to distinct cleavage sites along the predicted fold-back region of the amiRchs1 precursor (Figure 3C), and most of these sites corresponded to the ends of small RNAs identified by deep sequencing. The two most frequent sites corresponded to the two high-intensity bands (bands 3 and 4). The distance between the cleavage sites was mainly 20-23 nt, which is consistent with the rule that DCL1 cleaves pri-miRNA at 21 nt intervals [15]. These results showed that the processing of the amiRchs1 precursor in petunia is consistent with the processing mechanism of the ath-miR319a precursor in *Arabidopsis*; thus, processing begins with a cleavage below the terminal loop, which is followed by three more DCL1 cleavages towards the base of the stem-loop structure of the precursor [19]. However, the accuracy of cleavage appears to be reduced and extra intermediates are produced.

### Mapping small RNAs to the amiRchs1 precursor

Small RNAs in amiRchs1-transgenic petunia petals were analyzed using the deep-sequencing technique. Sequencing of the small RNA library produced a total of 11,076,276 Illumina reads from 603–8 petals. Following quality control and the removal of non-miRNA sequences representing other RNA species such as rRNA, tRNA and snRNA, 9,387,095 high-quality reads were obtained, ranging from 18 nt to 32 nt in length. The sequences ranged mainly from 19 nt to 25 nt in size, with two peaks at 21 nt and 24 nt (Figure S2). After collapsing identical sequences, 2,661,170 unique clones were extracted. Unless otherwise indicated, only those clones with more than five sequencing reads were used in the analysis below, in order to reduce the potential for the introduction of sequencing errors.

**Figure 4:**
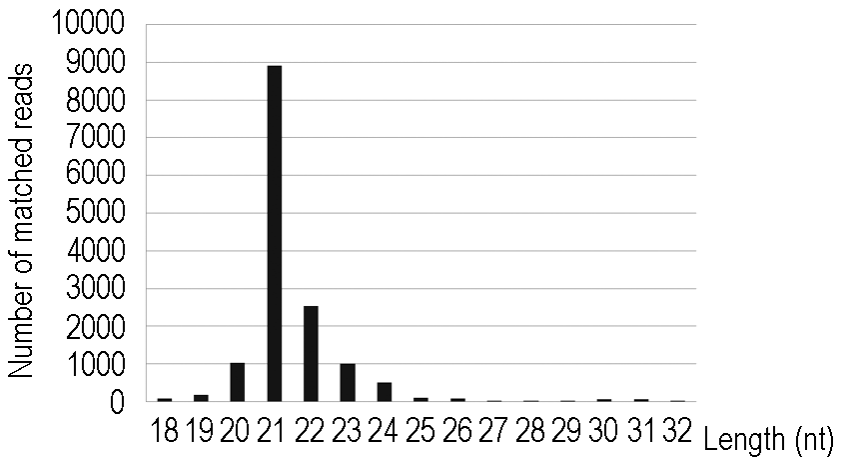
Size distribution of small RNAs mapped to the amiRchs1 precursor.

Amongst the total of 14,668 reads that matched the predicted amiRchs1 precursor in transgenic petals, two prominent peaks were observed, of 21 nt (especially) and 22 nt, respectively (Figure 4). This was consistent with the fact that plant microRNAs are processed by DCL1 or DCL4 [47]. A unique small RNA sequence was isolated, based on 3,885 reads of the predicted amiRchs1 sequence. Of all the sequences that matched the amiRchs1 precursor, this was the most frequently occurring, indicating that the synthesized microRNA precursor functioned effectively in transgenic petunia.

**Figure 5:**
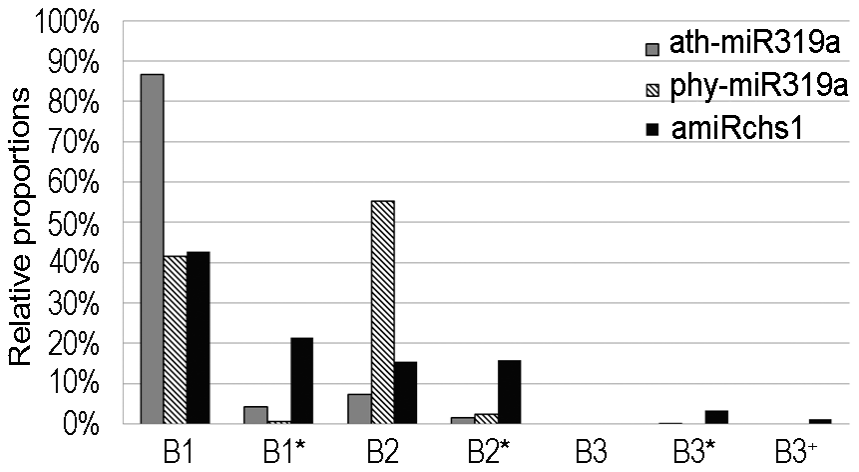
Relative proportions of small RNAs arising from different regions along the miR319 precursors. The regions are the same as indicated in Figure 3.

The position and abundance of the small RNAs that mapped to the amiRchs1 precursor are shown in Figure 3B and Figure 5. For comparison, small RNA deep-sequencing data for *Arabidopsis MIR319a* were retrieved from the miRBase database (v20, http://miRbase.org/index.shtml), and incorporated into the ath-miR319a precursor. According to our results and based also on previous studies [19], [48], [49], we defined seven blocks of sequence along the miR319a precursor (Figure 3A and B). The most abundant sequence reads within each block were taken as the representative sequence for the block as a whole. All unique sequences having more than a 15 nt overlap with the representative sequence, and extending for no more than 6 nt beyond the representative sequence at either end, were considered to belong to the same block. Thus, Block1 (B1) corresponded to miR319a.1 (guide strand) region, and B1^*^ to miR319a.1^*^ (passenger strand); B2 corresponded to miR319a.2, and B2^*^ to miR319 a.2^*^; B3 corresponded to the region between B1 and B2, and B3^*^ to the region between B1^*^ to B2^*^; and finally B3^+^ corresponded to a region longer than B3, in which the 3′ ends of B3 sequences stretched into the middle of B1.

More than 86% of the small RNA sequences originating from the ath-miR319a precursor matched the B1 region, and those that matched the B1^*^, B2, B2^*^ and B3^*^ regions occurred at low frequency (Figure 3A and Figure 5). By contrast, microRNA sequences arising from the B1 region of the amiRchs1 precursor accounted for only 43% of the total, with a concomitant increase in the relative proportions of small RNA sequences from other regions (Figure 3B and Figure 5). Although no sequences belonging to the B3 region were retrieved when deep-sequencing data for ath-*MIR319a* from miRBase were used, Bologna et al. [19] had previously identified B3-region small RNAs in another publicly available database. Therefore, a search of the deep-sequencing data was undertaken for small RNA clones that belonged to the B3 region and were represented by fewer than five reads. Seven unique sequences, with a total of 11 reads, were identified that belonged to the B3 region, indicating that B3-region small RNAs accumulated at a low level. A few B3^+^ small RNA sequences that overlapped with the B3-region sequences were also identified; these had 3′ ends extending into the central regions of B1 small RNAs, so that they were longer than the B3-region small RNAs (Figure 3B and Figure 5). These deep-sequencing results indicated that processing of the amiRchs1 precursor in petunia produced small RNAs that arose from the same regions as the small RNAs produced from the processing of the ath-miR319a precursor in *Arabidopsis*, but that the relative proportions of the small RNAs derived from the different regions were altered, and that abundant unwanted small RNAs were produced.

To investigate whether this production of additional small RNAs from the amiRchs1 precursor could in fact have arisen through the processing of petunia miR319 homologue precursors, deep-sequencing data for: i) wild-type petunia (GSM433598 and http://www.petunia_smrna.leeds.ac.uk/), and ii) line V26 with co-suppressed *CHS* genes (GSM346607), were searched using the amiRchs1 precursor sequence. No sequences were identified that showed extensive complementarity to the additional small RNAs. Highly accumulated sequences (≥5 reads) that were complementary to amiRchs1 (which is complementary to petunia *CHS*) were, however, found in GSM346607. Secondly, the GenBank databases were searched using the ath-miR319a (identical to ath-miR319b) sequence to determine whether petunia miR319 precursors could be found. From the petunia EST database, FN011712 was found, containing a fragment showing perfect complementarity to miR319a. Analysis of the secondary structure of FN011712 mRNA using Mfold [43] showed that it encoded a stem-loop region similar to that of the miR319a precursor (Figure 3D), indicating that FN011712 represented a homologue of *MIR319*. Since it encoded a mature microRNA identical to ath-miR319a and ath-miR319b, but the secondary structure of its precursor was more similar to that of the ath-*MIR319a* precursor than to that of the ath-*MIR319b* precursor (Figure S3), it was therefore designated *phy-MIR319a*. We next searched our deep-sequencing data for small RNA sequences that originated from the phy-miR319a precursor and incorporated them into its stem-loop structure (Figure 3D). This revealed that these small RNAs were concentrated within the B1, B1^*^, B2 and B2^*^ regions, and that the relative proportions of small RNAs that originated from these regions were different from the corresponding relative proportions observed in the case of the ath-miR319a precursor (Figure 5). Because the sequence of the phy-miR319a precursor differed from that of the amiRchs1 precursor, it was unlikely that the processing of petunia miR319 precursors would have contributed to the observed accumulation of additional small RNAs showing sequence homology to the amiRchs1 precursor. In addition, using all the undesired small RNA sequences identified in this study, a search was undertaken for microRNA hairpin precursor sequences in all the organisms deposited in miRBase (v20). Sequences showing 100% identity were only found from *Arabidopsis thaliana* and *Arabidopsis lyrata*; therefore, the possibility was minimal that these undesired small RNA sequences were coincidentally produced from other petunia miR319 precursors that have not been identified.

### Perfect pairing between amiRchs1 and its *CHS-J* target did not induce secondary small RNAs

A previous study had suggested that perfect pairing between a microRNA and its target would prime the biogenesis of secondary small RNAs [50], and it had been suggested that three mismatches to their target genes should be deliberately introduced into the 3′ regions of amiRNAs so as to reduce the likelihood that amiRNAs would trigger the production of secondary RNAi [24]. Amplification of an initial amiRNA signal by secondary small RNAs has also been observed in *Physcomitrella patens* [34]. The sequence of amiRchs1 matched that of *CHS-J* (X14597) perfectly between nt2661 and nt2681, and we therefore searched our deep sequencing data for small RNA sequences that showed matches to *CHS-J* and *CHS-A* transcripts. With the exception of the amiRchs1 sequence, no highly accumulated small RNA sequence (≥5 reads) showing extensive complementarity to *CHS-A* or *CHS-J* transcripts was found, indicating that the amiRchs1 guided target cleavage did not induce the biogenesis of phasiRNAs. This result was also confirmed by the 5′-RLM-RACE results: only one clear band was detected for *CHS-A* and *CHS-J*, respectively (Figure 2C). Interestingly, from our deep sequencing data and from GSM433598, we identified a few unique sequences that matched the 5′ promoter sequence or the intron region of *CHS-A* (Table S1).

### Small RNAs displaying tailing modification

From the deep-sequencing data of 603-8 transgenic petals, we also identified a class of small RNAs that could each be divided into two parts: a 5′ genome-matched component (5GMC) [51], which matched perfectly to the amiRchs1 precursor, and a 3′ “tail” component. The 5GMC sequences were between 17nt and 31 nt long, and their tails were mostly 1 nt, with the longest being 3 nt; the sequences arising from B1, B1^*^, B2 and B3^+^ comprised 116, 142, 311 and 12 reads, respectively (Table 2). The most frequently occurring “tail” nucleotide was uridine (45%), followed by adenine (28%), cytosine (16%) and guanine (11%). Because the 5GMC sequences of these small RNAs corresponded to the regions from which previously-identified small RNAs arising and they accumulated at relatively high levels, we concluded that they were tailed variants of the small RNAs arising from the amiRchs1 precursor. We also identified tailed small RNA variants arising from the phy-miR319a precursor; however, these originated only from B1 (501 reads) and B1^*^ (23 reads). Their tails were 1–2 nt long, consisting predominantly of uridine (84%), with smaller proportions of cytosine (14%) and guanine (2%) (Table 3).

**Table 2:** Small RNAS arising from the amiRchs1 precursor with 3′ tail.

Unique ID	Sequence (3′ tail nucleotides are capitalized)	Reads	Position	Total reads
4529255	tgttggtacatcatgagtcg**T**	28	B1	116
127628	tgttggtacatcatgagtcgc**A**	23	B1	
5944084	tgttggtacatcatgagtcgc**C**	18	B1	
4124961	tgttggtacatcatgagtcgctct**T**	10	B1	
4831892	tgttggtacatcatgagtcgct**T**	10	B1	
1311807	tgttggtacatcatgagtc**T**	9	B1	
2744001	tgttggtacatcatgagtcgctct**A**	8	B1	
4085913	tgttggtacatcatgagtcg**TTC**	5	B1	
5323907	tgttggtacatcatgagtcgc**CT**	5	B1	
5665375	aactcatgatgaaccaacttc**T**	41	B1^*^	142
1874545	—ctcatgatgaaccaacttcac**T**	27	B1^*^	
927297	---tcatgatgaaccaacttcac**T**	46	B1^*^	
4528246	----catgatgaaccaacttc**T**	28	B1^*^	
4270474	aatgaatgatgcggtagacaaa**A**	103	B2	311
4389442	aatgaatgatgcggtagacaaat**C**	48	B2	
5577805	aatgaatgatgcggtagacaaat**G**	47	B2	
1049979	aatgaatgatgcggtagacaaa**C**	32	B2	
5744117	aatgaatgatgcggtagacaaat**A**	22	B2	
3006666	aatgaatgatgcggtagacaa**T**	12	B2	
5142252	aatgaatgatgcggtagac**T**	10	B2	
5674106	aatgaatgatgcggtagacaa**TT**	9	B2	
6175468	aatgaatgatgcggtagacaaa**G**	9	B2	
4479594	aatgaatgatgcggtagaca**T**	7	B2	
1992832	aatgaatgatgcggtagacaa**G**	6	B2	
3318766	aatgaatgatgcggtagacaaa**AC**	6	B2	
3095025	ttggatcattgattctctttgatgttggtac**T**	7	B3^+^	12
1404229	ttggatcattgattctctttgatgttggtac**C**	5	B3^+^	

**Table 3:** Small RNAs arising from the phy-miR319a precursor with 3′ tail.

Unique ID	Sequence (3′ tail nucleotides are capitalized)	Reads	Position	Total reads
1326087	-ttggactgaagggagctcc**TT**	112	B1	501
2403353	cttggactgaagggagctcc**T**	95	B1	
6094764	cttggactgaagggagctcc**TT**	80	B1	
2661774	-ttggactgaagggagctccct**T**	73	B1	
4673852	cttggactgaagggagctccc**C**	54	B1	
3325799	-ttggactgaagggagctccc**C**	40	B1	
1418357	-ttggactgaagggagctcc**T**	36	B1	
4245887	cttggactgaagggagctccct**T**	11	B1	
1975016	attcaacgatgcatgagctg**G**	12	B1^*^	23
4917471	attcaacgatgcatgagctg**C**	6	B1^*^	
2934896	attcaacgatgcatgagctgt**T**	5	B1^*^	

### Potential targets found by target search

**Figure 6:**
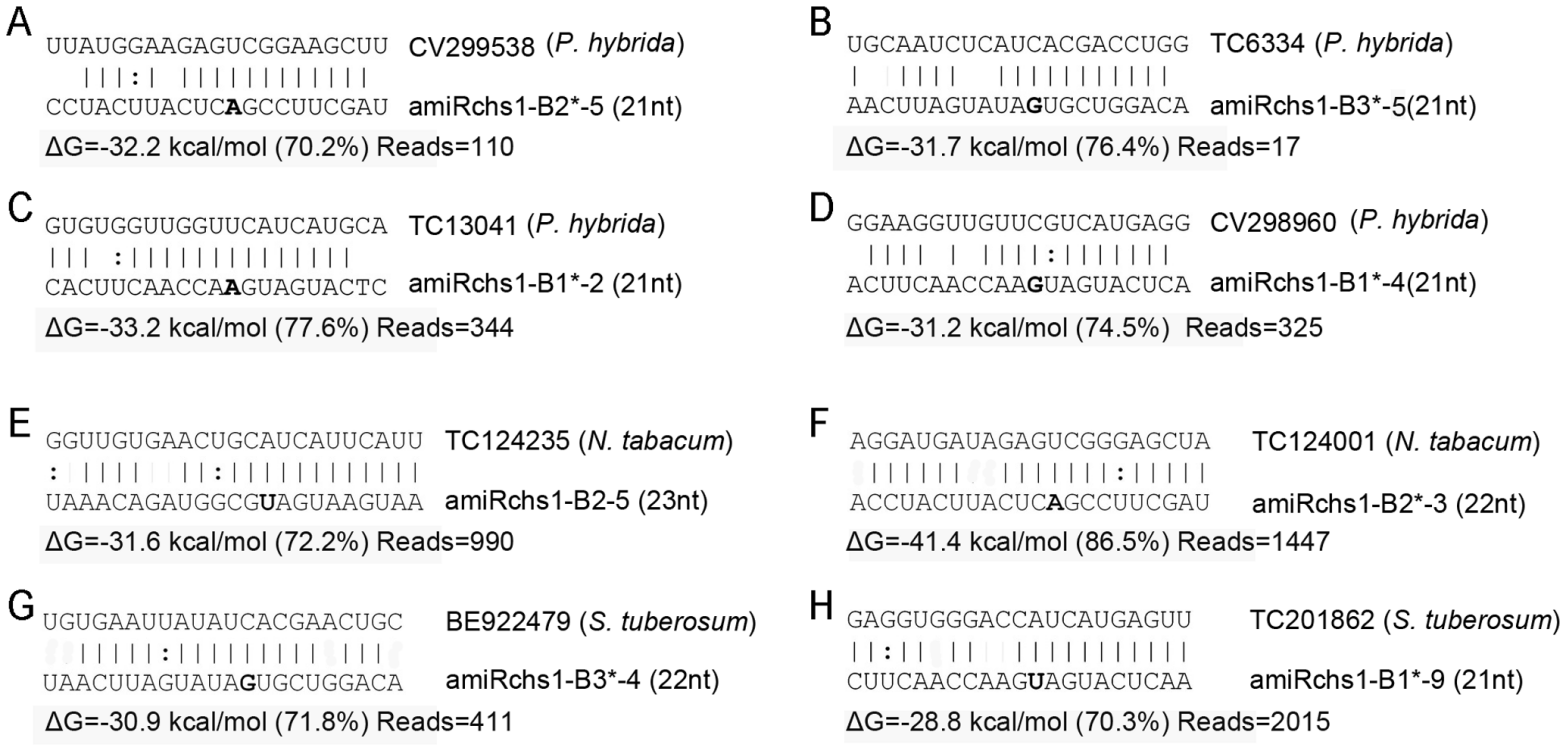
Alignments of selected small RNAs and some of their potential mRNA targets. Included are small RNA length, hybridization energy, percentage of free energy compared to a perfectly complementary target and small RNA reads identified in this study.

A target search using default parameters found 72 potential target sequences in the *Petunia* genus (Table S2). Since different sequence IDs in the PlantGDB (http://www.plantgdb.org/) and GeneIndex (http://compbio.dfci.harvard.edu/tgi/) databases may represent the same transcript sequence, in reality the total number should be lower than 72. Nevertheless, this analysis indicated the existence of potential targets in *Petunia* transcripts. Alignments between a small number of selected 21 nt small RNAs and some of their potential mRNA targets in *Petunia hybrida* are shown in Figure 6. When pairing with CV299538, amiRchs1-B2*-5 (Figure 6A) met the required criteria for amiRNA design [24]. The parameters of the other three pairs presented in Figure 6B, C and D met the (less stringent) criteria for plant microRNA target selection proposed by Schwab et al. [52]. These criteria include the empirical parameters for target recognition: i.e. no mismatch at claevage site (positions 10 and 11); no more than one mismatch at positions 2-12; no more than 4 mismatches downstream of position 13 and no more than two in a row; low overall free energy of targets paired with their corresponding miRNAs (at least 70% compared with a perfect match) [24], [52]. Many other transcripts from the *Petunia* genus shown in Table S2 were also potential targets and met these criteria for target selection.

Since genomic information for petunia is very limited, we searched the genomes of tobacco, tomato and potato to determine whether potential targets would be found if the same collection of undesired small RNAs were to be produced in these plant species. There were 61, 33 and 39 potential targets identified from tobacco (GeneIndex NtGI7.0), tomato (LGI-13.0) and potato (StGI-13.0), respectively (Table S2). In a few cases, transcripts were potential targets for overlapping small RNAs. For example, all of the tobacco ESTs TC123041, TC126640, TC164324 and TC124235 were potential targets for all of the overlapping small RNAs amiRchs1-B2-4 (24nt), B2-5 (23nt), B2-6 (22nt), B2-7 (21nt) and B2-8 (20nt) (Table S2). Alignments between the most abundant small RNA from each block (B2, B2^*^, B3^*^ and B1^*^) and some of their mRNA targets are shown in Figure 6E–H. These results indicated that if additional small RNAs were to be produced in other Solanaceae plants besides petunia, they might similarly have potential detrimental functions.

## Discussion

An artificial microRNA was designed for the silencing of petunia *CHS* genes, following the rules set by Schwab et al. [24]. When the synthesized amiRchs1 precursor was constitutively expressed in petunia, the resulting transgenic flowers showed the *CHS* gene-silencing phenotype, the predicted artificial microRNA was accumulated preferentially, the *CHS-A* and *CHS-J* genes were each cleaved exactly at the predicted site, and the accumulation of *CHS-A* and *CH-J* full-length transcripts was reduced. These results indicated that the amiRchs1 functioned as expected and demonstrated that artificial microRNA technology based on *Arabidopsis* microRNA precursors could be used successfully in *Petunia hybrida*.

On the other hand, high levels of unwanted, additional miRNA-like RNAs accumulated. Three factors may have contributed to this unexpected phenomenon. First, the replacement of the natural miR319a by the engineered amiRchs1 may have led to changes in the structural features of the miRNA precursors, with implications for the accuracy of cleavage and for the modification and the stability of the cleavage products. An extra-base pair was introduced below the miRNA/miRNA^*^ duplex (Figure 3A and B) when the amiRchs1 was synthesized using the procedure of Schwab et al [44]. This extra-base pair may influence the processing of amiRNA precursors. However, since miR319 precursors are processed from loop to base, and the precursor sequences below the miRNA/miRNA^*^ duplex are dispensable [19], the role of this extra-base pair would be expected to be limited. Secondly, there may conceivably exist subtle differences between species in the machinery of miRNA processing. It was observed that the B2-region small RNAs arising from the phy-miR319a precursor accumulated at higher frequency than those originating from the ath-miR319a precursor (Figure 5). This difference could, however, have resulted from structural differences between the miRNA precursors. It is therefore not possible at present to make a distinction between the possible effects of structural differences and species differences. Thirdly, overexpression of the amiRchs1 precursor may have overwhelmed the endogenous microRNA processing machinery, thereby compromising the accuracy of processing. At present, our knowledge of the processing of artificial microRNA precursors is limited. Even with the use of carefully selected amiRNAs designed with the use of WMD, the success of amiRNA-based gene silencing only approached 75% [44]. No method has yet been developed for predicting whether additional small RNAs will be produced in large quantities. Therefore, at least under some conditions, when artificial microRNA technology is used to suppress gene expression, high levels of additional small RNAs will be produced.

Derivatives of artificial microRNAs and extra miRNA-like RNAs with modified “tails” were identified from small RNAs of 603–8 transgenic petals. Since ‘the 3′ truncation and tailing take place while miRNAs are in association with ARGONAUTE1 (AGO1), either during or after RISC assembly' [53], these tailed small RNAs found in transgenic petals indicated that the additional small RNAs could be in association with AGO proteins. As miRNAs are loaded onto AGO proteins to silence target genes, these small RNAs may be turned into components of active RISCs, and play a role in regulating gene expression. Possible target genes of miR319b.2 small RNAs have been identified in the *Arabidopsis* genome [49]. On account of the high complexity of most plant genomes (and given that the genomes of most plants are more complex than that of *Arabidopsis*), it is possible that target genes for these additional small RNAs may exist in a number of plant species. Using TargetSearch integrated in WMD3 to explore possible targets of small RNAs, potential targets of B2, B1^*^, B2^*^ and B3^*^ small RNAs were discovered in petunia and other Solanaceae plants (Figure 6, Table S2). For many transcripts, the parameters of pairing with these undesired small RNAs were found to meet the criteria for plant microRNA target selection (Figure 6, Table S2) [52]. If undesired small RNAs are produced only at low frequency, their influence may be negligible; if, on the other hand, they are likely to be accumulated to high levels, it is desirable to take into account their potential functions and effects. In using artificial microRNA gene-silencing technology in plants, it must be recognized that the biogenesis of artificial microRNAs may generate additional small RNAs with the potential to affect unintended targets. One consequence of this is that the phenotypic consequences of the expression of amiRNAs require very careful interpretation.

The initial design rules for the generation of artificial microRNAs stipulated a mismatch within the amiRNA/target duplex at the 3′ end of the amiRNA in order to avoid the production of secondary small RNAs [24], yet the results presented here indicated that perfect pairing between amiRchs1 and *CHS-J* transcript did not result in the biogenesis of phasiRNAs. Recent studies have shown that the structural determinant for plant phasiRNA production is not in fact perfect pairing between a microRNA and its target, but the presence of asymmetrically positioned, “bulged” bases in the miRNA/miRNA^*^ duplex [54]. The results of the present study are consistent with this model. Park et al. [55] reported that the use of an artificial miRNA having perfect complementarity to its target gene achieved highly efficient gene silencing; similarly, amiRNAs designed to have perfect complementarity to viral gene targets have shown high specificity [56]. Thus, perfect complementarity to the target can in future be incorporated into the design of amiRNAs, in order to increase the specificity and efficiency of amiRNA-induced gene silencing.

## Supporting Information

Figure S1 **Phenotype of amiRchs1 transgenic flowers from ‘GL8’ and ‘10V1’ genotypes.**
(TIF)

Figure S2 **Size distribution of small RNA clones from amiRchs1 transgenic petals.**
(TIF)

Figure S3 **Alignment of mature miR319 sequences and the fold-back structures of miR319 precursors.**
(TIF)

Table S1 Small RNAs mapped to the promoter and intron regions of *CHS-A* (X14591).
(XLSX)

Table S2 Potential mRNA targets of the undesired small RNAs in petunia, tobacco, tomato and potato.
(XLSX)
