# Supplementary figures and images for: Undesired Small RNAs Originate from an Artificial microRNA Precursor in Transgenic Petunia (Petunia hybrida)
Source: PLoS One. 2014 Jun 4;9(6):e98783. doi: 10.1371/journal.pone.0098783 (PMC4045805; doi:10.1371/journal.pone.0098783)

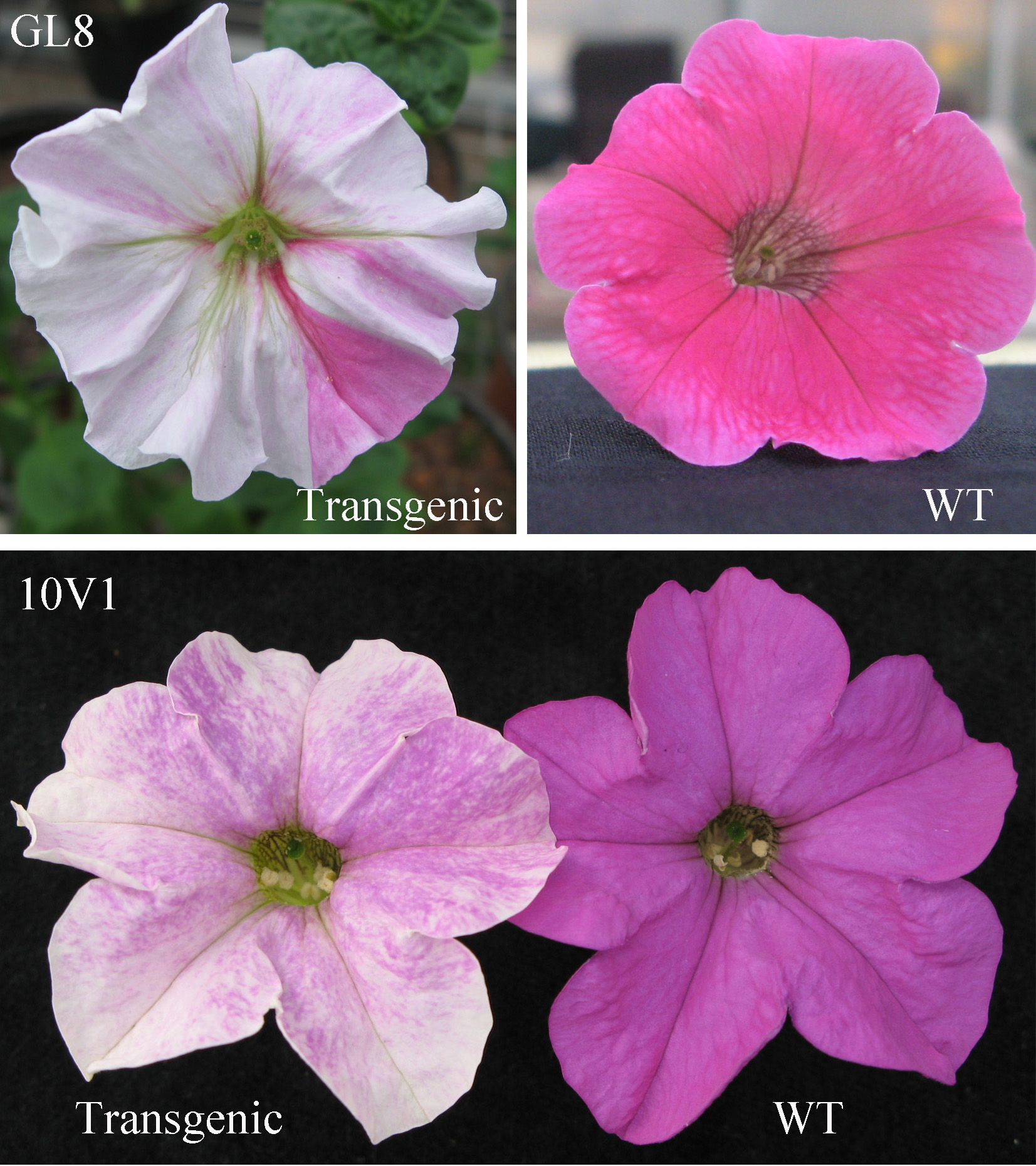

Supplement: Figure S1 — Phenotype of amiRchs1 transgenic flowers from ‘GL8’ and ‘10V1’ genotypes. (TIF) [file pone.0098783.s001.tif]

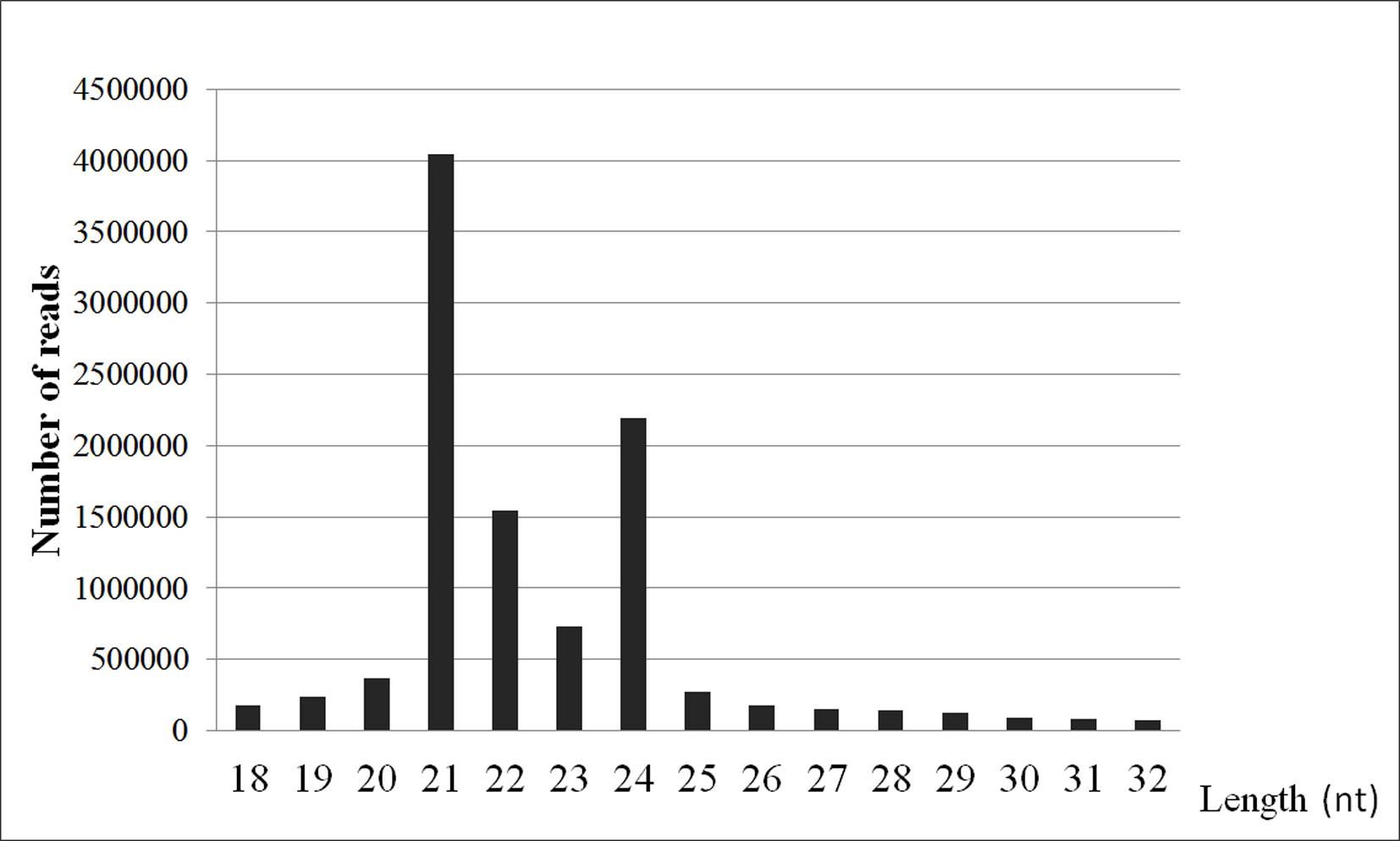

Supplement: Figure S2 — Size distribution of small RNA clones from amiRchs1 transgenic petals. (TIF) [file pone.0098783.s002.tif]

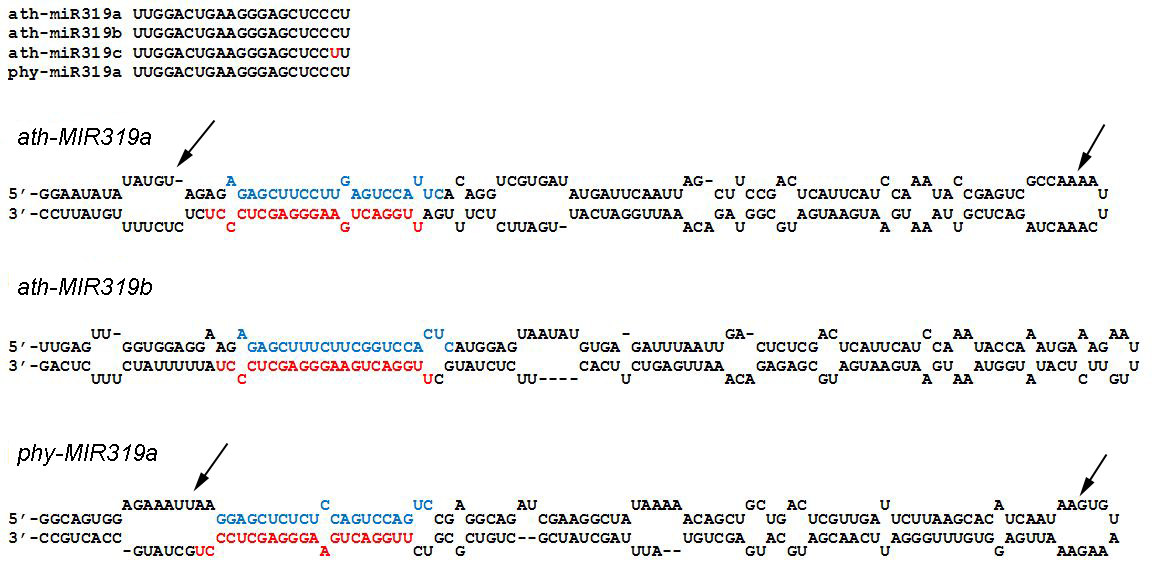

Supplement: Figure S3 — Alignment of mature miR319 sequences and the fold-back structures of miR319 precursors. (TIF) [file pone.0098783.s003.tif]
